# Slime molds (Myxomycetes) causing a “disease” in crop plants and cultivated mushrooms

**DOI:** 10.3389/fpls.2024.1411231

**Published:** 2024-06-10

**Authors:** Zhaojuan Zhang, Chao Zhai, Yu Li, Steven L. Stephenson, Pu Liu

**Affiliations:** ^1^ Engineering Research Center of Edible and Medicinal Fungi, Ministry of Education, Jilin Agricultural University, Changchun, China; ^2^ Department of Biological Sciences, University of Arkansas, Fayetteville, AR, United States

**Keywords:** plasmodial slime molds, crop health, colonization, mushroom crop, cultural control, chemical control

## Abstract

Myxomycetes (plasmodial slime molds) are eukaryotic protist predators that are associated with wood, leaf litter, and soil in forests, where they feed on bacteria, protozoans, and (to a more limited extent) fungi. The health of crop plants is essential because they represent a primary food source for humans. However, when myxomycetes produce numerous fruiting bodies on the stems and leaves of crop plants, which is herein referred to as a myxomycete colonization, this has the potential of interfering with plant photosynthesis, transpiration and respiration by blocking out light and covering stomata. Myxomycetes are not pathogens, but their occurrence on plants can be mistakenly interpreted as some type of infection. However, this phenomenon has been largely ignored. This paper provides a comprehensive overview of the taxonomic and economic diversity of the organisms involved in myxomycete colonization. In addition, the various types of myxomycete colonization reported in the literature are described and discussed, a number of images provided, and cultural and chemical prevention and control measures are summarized. The latter should be of significant relevance for local production of crops and plant protective stations. While myxomycetes are not pathogens of crop plants, some species can seriously impact commercially grown mushrooms. Reports of myxomycetes affecting mushrooms are also described in this paper.

## 1 Introduction

The health of crop plants has major public implications when farmers are able to access basic crop healthcare and services from relevant authorities to evaluate infested or suspected infested crop plants. By extension, this is also advantageous for human health (Vega et al., 2020; Jia et al., 2023). Over the past few decades, people-crop plant studies have increasingly focused on empirically demonstrating relationships between crop plants and health (Grandin, 2022). There is a vast array of diseases in the natural environment of crop plants, such as fungal diseases (Ceasar and Ignacimuthu, 2012; Wang, 2023), bacterial diseases (Hikichi, 2016; Li et al., 2023), viral diseases (Bhat et al., 2022; Zhang et al., 2022), and nematode-caused diseases (Kaloshian and Teixeira, 2019; Bhat et al., 2023). These crop plant diseases constitute a huge economic and environmental threat to agricultural and forestry production. However, there is increasing difficulty in identifying new plant diseases and what mistakingly appear as plant diseases as a result of ongoing environmental change (Jones, 2016; Jain et al., 2019). There is a need to identify the factors influencing the emergence and the increasing incidences of these diseases.

The myxomycetes (true slime molds or plasmodial slime molds) are a monophyletic taxon within the phylum Amoebozoa as the class Myxomycetes or Myxogastrea (Adl et al., 2012; Kang et al., 2017b). These organisms have a peculiar life cycle that encompasses a microscopic amoeboflagellates (the first tropic stage), a multinuclearte plasmodium (the second trophic stage), and a macroscopic fruiting body (the reproductive stage) within which spores are produced (Everhart and Keller, 2008; Stephenson and Feest, 2012; Stephenson and Rojas, 2017). Like many other protist predators, myxomycetes feed on bacteria and other microorganisms. As such, they represent an ecologically important component of terrestrial nutrient cycles (Walker et al., 2019). Well known microhabitats for myxomycetes include decaying wood (Fukasawa et al., 2018), aerial plant litter, ground plant litter (Pecundo et al., 2017), the bark of living and dead trees, and dung (Abdel-Raheem, 2002). They even occur on the inflorescences of Neotropical herbs (Schnittler and Stephenson, 2002) and in aquatic habitats (Lindley et al., 2007).

**Figure 1:**
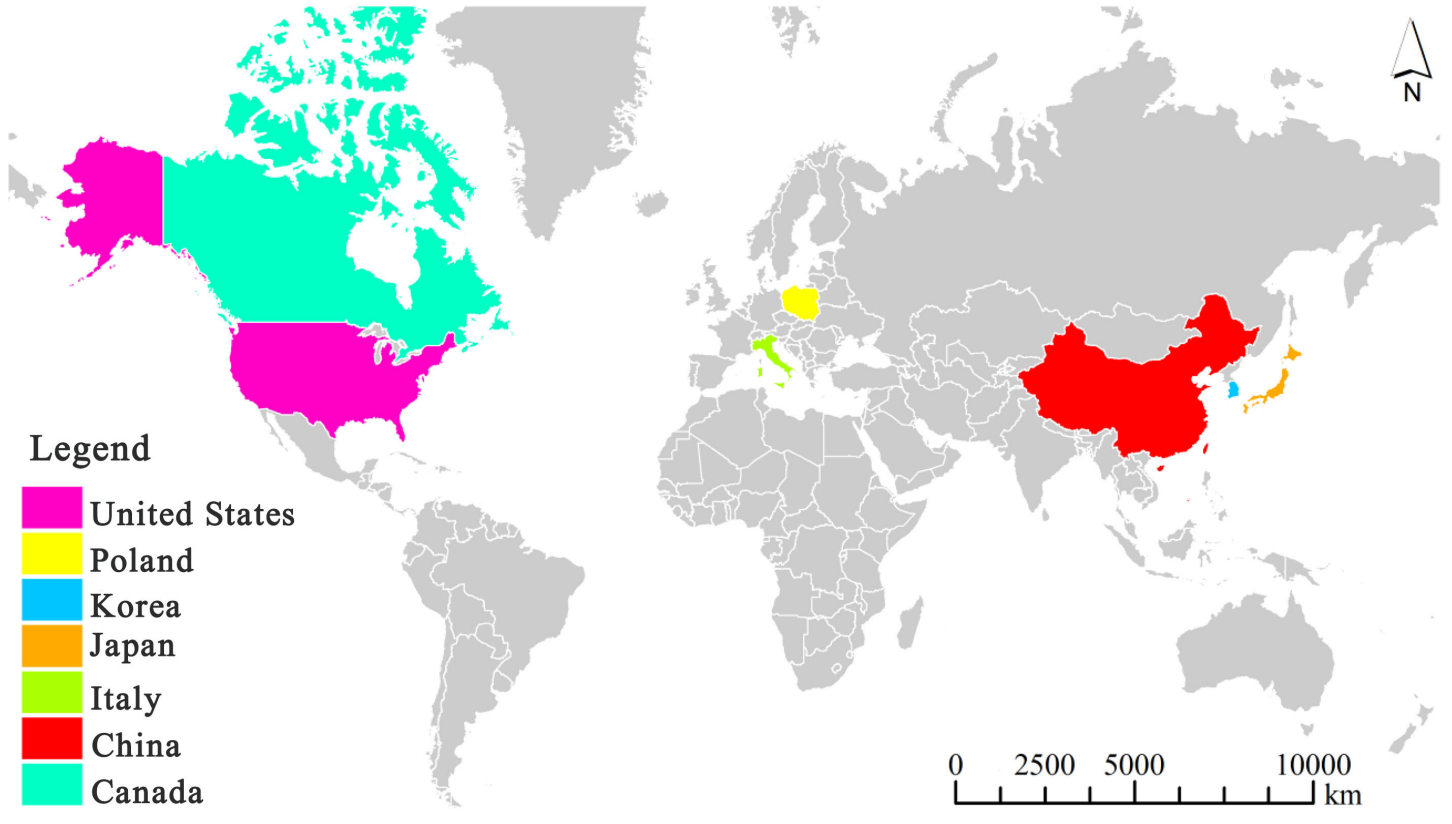
A map showing countries reporting myxomycete colonization by December 2023. Note: the map was drawn using ArcGIS software by summarizing the countries where the distribution of myxomycete colonization from **Table 1**, as shown in **Table 1** for references.

It is worth noting that myxomycetes have been reported as a common crop plant “diseases” despite the fact that they are not pathogenic. Su (Su, 1987) observed the occurrence of myxomycetes on sweet potato seedlings over a period of six years, and considered this as a “disease”. Li (Li et al., 1994) reported a new slime mold “disease” on strawberries. Also, Couch (Couch, 1995) used the term “disease” for myxomycetes covering plant leaves. Herein, we refer to a myxomycete colonization as simply referring to the occurrence of myxomycetes, typically the fruiting bodies, on crop plants but not implying that myxomycetes are in any sense true pathogens. Reports of this phenomenon come from seven countries throughout the world, with China having the most widespread instances of myxomycete colonization (**Figure 1**). These reports include affected grain crops (Su, 1987; Kim et al., 2007; Wang et al., 2014; Chen et al., 2021), cash crops (Mortensen and Molloy, 1989; Li et al., 1992; Guan, 2004; Kong et al., 2014; Wang et al., 2014; Tu et al., 2016; Wang et al., 2016; Xie et al., 2017; Huang, 2019; Chen et al., 2021), vegetable crops (Wang and Pan, 1985; Kim et al., 2009; Crescenzi et al., 2015), fruit crops (Golenia and Rebendel, 1970; Filipowicz, 1979; Li et al., 1994) and ornamental crops (Zhang and Li, 2003; Zhao et al., 2010) (**Table 2**). According to the statistics compiled from an intensive search of the published literature, a total of 31 crop plants have been reported to have “slime mold disease”. Among these, cash crops account for the highest proportion, with 17 crop species associated with myxomycetes, accounting for 54.8% of all crop plant types.

**Table 1:** The effects of myxomycete colonization on five different types of major crops.

Affected crops	Geographic distribution	Disease-causing myxomycetes	Disease symptoms	Images	Harm incurred	References
Grain crops						
Sweet potato [*Ipomoea batatas* (L.) Lam.]	Daejeon, Korea United States	*Fuligo septica* *Fuligo violacea* *Physarum cinereum*	Produces fruiting bodies on stems, leaves and petioles of sweet potato and appeared as grayish brown dust on the sweet potato plants	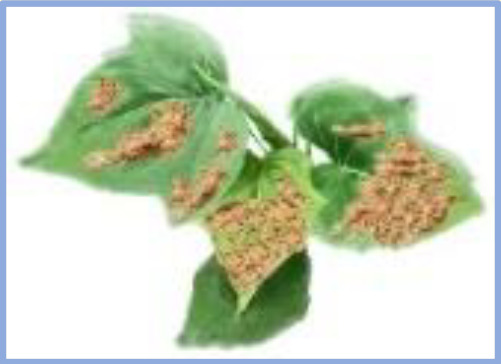	*——*	(Clark and Moyer, 1988; Farr et al., 1989; Cho and Shin, 2004; Kim et al., 2007)
	Daejeon, Korea	*Stemonitis herbatica*	Produces fruiting bodies on stems, leaves and petioles of sweet potato and appeared as dark brown dust on the sweet potato plants	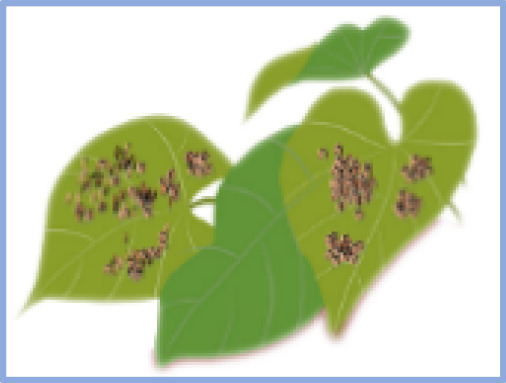	*——*	(Kim et al., 2007)
	Turpan, Xinjiang, China Japan	*Fuligo gyrosa*	Covered with stained white or brown gelatin of stems and leaves, later dense gray or black fruiting bodies	*——*	Inability to photosynthesis Plants stunted and shrink, losing cultivation value	(Su, 1987; The Phytopathological Society of Japan, 2000)
Foxtail millet [*Setaria italica* (L.) P. Beauv.] Corn [*Zea mays* L.] Wheat [*Triticum aestivum* L.]	Nanyang, Henan, China	*Fuligo gyrosa* *Physarum cinereum*	Covered with black granular or powdered of stem and leaves, then white fruiting bodies	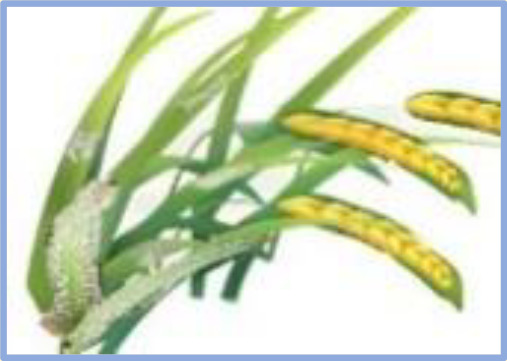	Inability to photosynthesis Plants stunted and shrink	(Wang et al., 2014; Chen et al., 2021)
Cash crops						
Peanut [*Arachis hypogaea* Linn.] Sesame [*Sesamum indicum* Linn.] Traditional Chinese medicine [*Acroptilon repens* (Linn.) DC.]	Nanyang, Henan, China Western Canada	*Fuligo gyrosa*	Covered with disk-shaped white, gray-white or brown foam plaques or protuberances on the back and margins of the leaves, with dense small black spots and scattered small black spots on the leaf surface	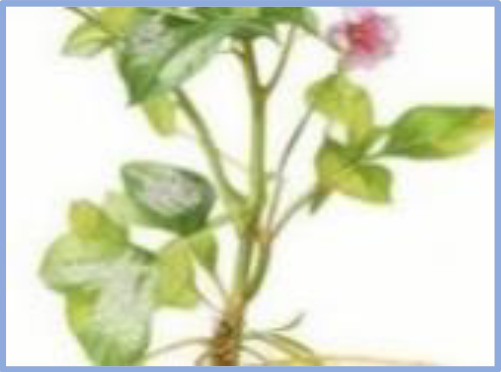	Inability to photosynthesis	(Mortensen and Molloy, 1989; Wang et al., 2014, Wang et al, 2016)
Dendrobe [*Dendrobium officinale* Kimura et Migo]	(Dongyang, Tiantai, Hangzhou, Leqing, Jinhua, Xinchang, Longquan, Pan’an, Lishui, Huangyan, Longyou), Zhejiang, China	*Fuligo septica*	Covered with different sizes and shapes aethalia of *D. candidum* plants, then formed black brown sorus after white thickened cortex of aethalia peeling off	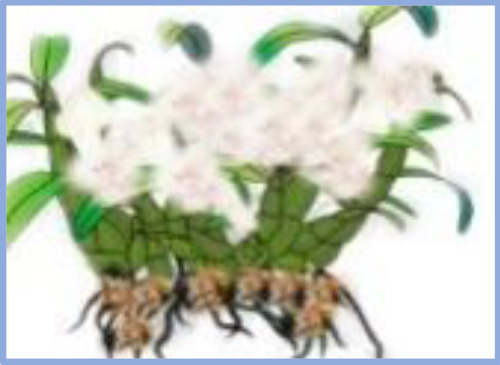	Affects photosynthesis and plant dwarfing	(Tu et al., 2016; Xie et al., 2017)
	Shaoxing, Zhejiang, China	*Physarum melleum*	Covered with stalked sporangia groups on young leaves of *D. candidum*, with broken spore case showing black spore mass intermingled with whitish capillitium	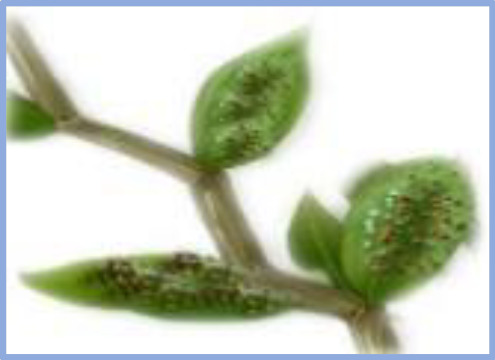	*——*	(Zhang et al., 2007)
	(Dongyang, Tiantai, Hangzhou, Leqing, Jinhua, Xinchang, Longquan, Pan’an, Lishui, Huangyan, Longyou), Zhejiang, China	*Comatricha pulchella*	First gray white granular material, then covered with black brown hair like protrusions (mature fruiting body)	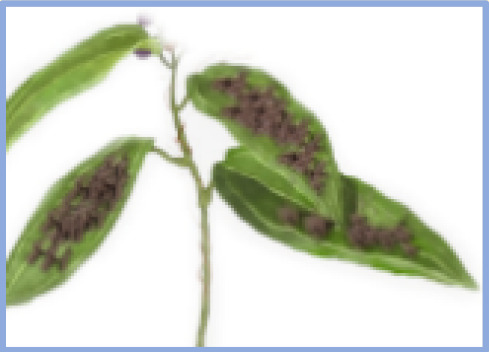	Affects photosynthesis and plant dwarfing	(Tu et al., 2016; Xie et al., 2017; Shen et al., 2021)
Mushroom [*Lentinula edodes* (Berk.) Pegler] *Auricularia* sp. [*Hericium erinaceus* (Bull.) Pers.]	Henan, China Baise District, Guangxi, China	*Stemonitis splendens* *Physarum* sp. *Tubifera* sp. *Physarum pezizoideum*	Hairy, black and shiny sporecystic stalk; protoplasm of obvious network; cylindrical spore sac, dense polymerization	*——*	Streaming ears, rotten, loss commodity value	(Li and Li, 1992; Lin, 1993; Chen et al., 2006)
[*Lentinula edodes* (Berk.) Pegler]	Changchun and Yanji, Jilin and Zhejiang, China Sanmenxia, Henan, China Geochang-gun and Gyeongnam, Korea	*Physarella oblonga* *Fuligo septica* *Physarum pezizoideum* *Physarum utriculare* *Physarum polycephalum* *Physarum melleum* *Craterium leucocephalum* *Stemonitis splendens* *Symphytocarpus longus* *Tubifera ferruginosa* *Lycogala epidendrum* *Arcyria denudata*	Covered with abundant bright yellow plasmodia in cultivation bags	——	The white mycelia became brown, thin and sparse, with inhibited growth leading to eventual death	(Chen et al., 2006; Lee et al., 2014; Li et al., 2017; Zhang et al., 2018)
Mushroom [*Auricularia heimuer* F. Wu, B.K. Cui & Y.C. Dai] [*Auricularia cornea* Ehrenb.]	Qinba Mountain, Shaanxi, China Mianyang, Sichuan, China	*Physarum pezizoideum* *Physarum utriculare* *Arcyria* sp.	lustered on the surface of piece of wood or *Auricularia heimuer*	——	Flowing ears	(He, 1991; Li et al., 1992)
Mushroom [*Cyclocybe aegerita* (V. Brig.) Vizzini]	Foshan, Guangdong, China	*Stemonitis herbatica*	Showed light yellow and white adhesive silk, fruiting bodies became soft and yellow	——	Rotten, fall down	(Guan, 2004)
Mushroom [*Agrocybe chaxingu* N.L. Huang]	Nanning, Guangxi, Sanming, Fujian, China	*Fuligo* sp. *Physarum* sp. *Stemonitis herbatica* *Stemonitis pallida*	Initially bright yellow or light yellow or milky white or yellow-green or gray-white, then the viscous turns into acne or jungle or hair	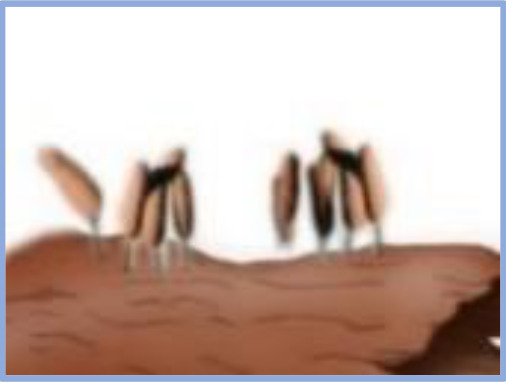	Fruiting body spoiled and rotten, difficult to form or even grow	(Liu and Lin, 2003; Liu, 2007; Chen, 2008)
Mushroom [*Grifola frondosa* (Dicks.) Gray]	Qingyuan, Lishui, Zhejiang, China	*Physarum galbeum*	Yellow plasmodia first migrated from the root of the fruiting body to the stem and then the pileus, then the affected parts became soft and putrid with slime on the surface	——	Reduced production	(Dai et al., 2023)
Mushroom (*Dictyophora* sp.)	Mount Wuyi and Sanming, Fujian, China	*Stemonitis fusca*	Dense dark purple-brown to dark red-brown fruiting bodies	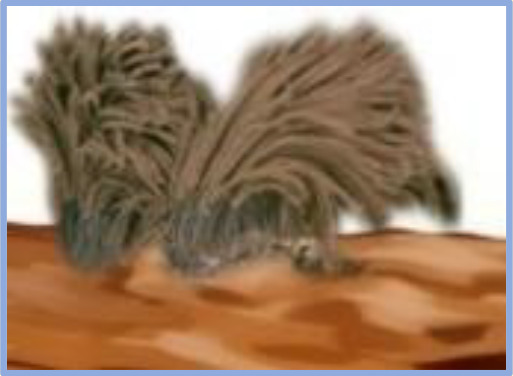	Fruiting body wet and rotting, the growth of mycelium inhibited or gradually extinct	(Liu, 2007; Liu et al., 2007; Bao, 2011)
Mushroom [*Pleurotus ostreatus* (Jacq.) P. Kumm.]	Mianyang, Sichuan, China	*Stemonitis* sp.	Densely clustered purple or brown fruiting bodies on the pileus and gill	——	——	(He, 1991)
Mushroom [*Schizophyllum commune* Fr.]	Wanan, Ji’an, Jiangxi, China	*Didymium bahiense*	Initially covered by brown, vein-like plasmodia. Plasmodia aggregated and produced numerous pale gray sporangia when the relative humidity dropped	——	Yield losses	(Hu et al., 2023)
Ginseng [*Panax ginseng* C. A. Meyer]	Hunjiang, Jilin, China	*Stemonitis herbatica*	Covered with large clusters of small clusters, cylindrical, blunt-headed, brown to reddish-brown fruiting bodies	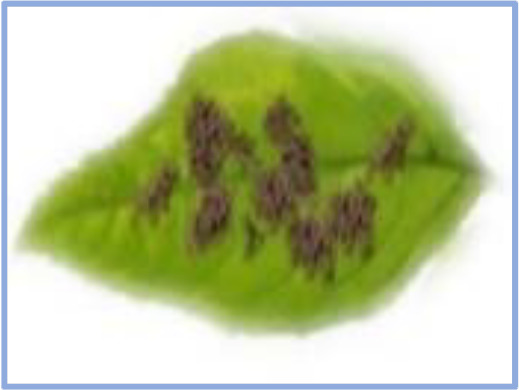	*——*	(Bai and Jiang, 1988)
Vegetable crops						
Lettuce [*Lactuca sativa* Linn.] Rocket [*Eruca sativa* L.] Chicory [*Cichorium endivia* Linn.] Celery [*Apium graveolens* Linn.]	Italy	*Physarum cinereum*	*——*	*——*	*——*	(Crescenzi et al., 2015)
Chilli [*Capsicum annuum* Linn.]	Huangshi, Hubei, China	*——*	Formed small light yellow particles on the front and back of the leaves, spread inward from the tip of the leaf and the edge of the leaf	*——*	Photoynthesis and respiratory obstruction	(Wang and Pan, 1985)
Fruit crops						
Oriental melon [*Cucumis melo* L. var. *makuwa* Makino] Watermelon [*Citrullus lanatus* (Thunb.) Matsum. et Nakai]	Chilgok, Korea Hubei, China	*Fuligo gyrosa* *Physarum cinereum*	Gray to dark gray fruiting bodies on the surface of stems, leaves, and petioles of the oriental melon plants	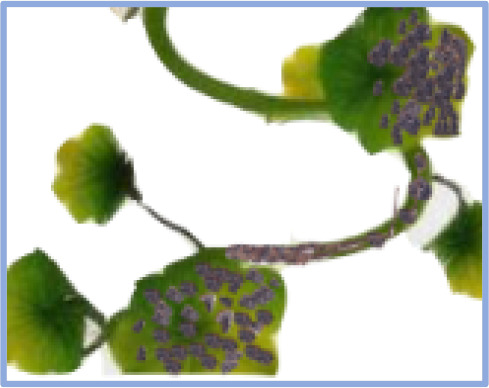	Severely affected plants were retarded in growth and later lower leaves blighted	(Zhao et al., 2010); Kim et al., 2009)
Strawberry [*Fragaria ananassa* Duch.]	Tonghua, Jilin, China Poland	*Diderma hemisphaericum* *Diachea leucopodia* *Physarum galbeum* *Diachea leucopodia*	Covered with white mold layer like a lime layer of leaves, stems and fruits, lesions on the leaves and stems turn brown	*——*	Fruit rotten	(Golenia and Rebendel, 1970; Filipowicz, 1979; Li et al., 1994)
Ornamental crops						
Ryegrass [*Lolium perenne* Linn.] Turfgrass (*Zoysia* spp.)	Jurong, Jiangsu, China	*Physarum cinereum*	Covered the gray-white fruit bodies in the leaves, petioles and leaf margins	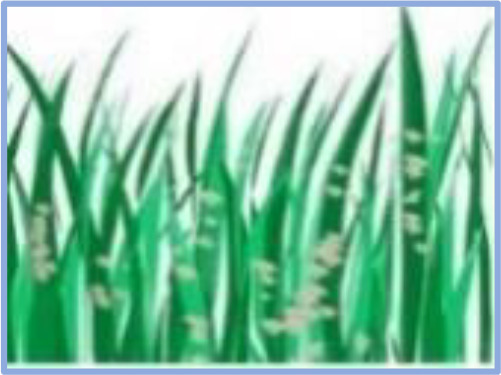	Photoynthesis and respiratory obstruction	(Zhang and Li, 2003; Zhao et al., 2010)

Format of geographic distribution: county/city name, province/state name, country name.

**Table 2:** Species of crop plants and mushrooms reported as affected by myxomycete colonization.

Grain crops		Cash crops			Vegetable crops	Fruit crops	Ornamental crops
Cereal crops	Tuber crops	Oil crops	Medicinal crops	Mushroom crops			
Foxtail millet [*Setaria italica* (L.) P. Beauv.] (Chen et al., 2021)	Sweet potato [*Ipomoea batatas* (L.) Lam.] (Su, 1987; Kim et al., 2007)	Peanut [*Arachis hypogaea* Linn.] (Wang et al., 2014, Wang et al, 2016)	Dendrobe [*Dendrobium officinale* Kimura et Migo] (Tu et al., 2016; Xie et al., 2017; Shen et al., 2021)	Mushroom [*Lentinula edodes* (Berk.) Pegler] (Li et al., 1992; Lin, 1993; Chen et al., 2006; Kong et al., 2014; Li et al., 2017)	Lettuce [*Lactuca sativa* Linn.] (Crescenzi et al., 2015)	Strawberry [*Fragaria ananassa* Duch.] (Golenia and Rebendel, 1970; Filipowicz, 1979; Li et al., 1994)	Ryegrass [*Lolium perenne* Linn.] (Zhao et al., 2010)
Corn [*Zea mays* L.] (Wang et al., 2014)		Sesame [*Sesamum indicum* Linn.] (Chen et al., 2021)	Traditional Chinese medicine [*Acroptilon repens* (Linn.) DC.] (Mortensen and Molloy, 1989)	Mushroom [*Agrocybe chaxingu* N.L. Huang] (Liu and Lin, 2003; Chen, 2008)	Rocket [*Eruca sativa* L.] (Crescenzi et al., 2015)	Watermelon [*Citrullus lanatus* (Thunb.) Matsum. et Nakai] (Zhao et al., 2010)	Turfgrass (*Zoysia* spp.) (Zhang and Li, 2003)
Wheat [*Triticum aestivum* L.] (Wang et al., 2014)			Ginseng [*Panax ginseng* C. A. Meyer] (Bai and Jiang, 1988)	Mushroom [*Cyclocybe aegerita* (V. Brig.) Vizzini] (Guan, 2004)	Chicory [*Cichorium endivia* Linn.] (Crescenzi et al., 2015)	Oriental melon [*Cucumis melo* L. var. *makuwa* Makino] (Kim et al., 2009)	
				Mushroom [*Auricularia heimuer* F. Wu, B.K. Cui & Y.C. Dai] (Li et al., 1992)	Celery [*Apium graveolens* Linn.] (Crescenzi et al., 2015)		
				Mushroom [*Grifola frondosa* (Dicks.) Gray] (Dai et al., 2023)	Chilli [*Capsicum annuum* Linn.] (Wang and Pan, 1985)		
				Mushroom (*Dictyophora* sp.) (Liu et al., 2007; Bao, 2011)			
				Mushroom [*Hericium erinaceus* (Bull.) Pers.] (Lin, 1993)			
				Mushroom [*Pleurotus ostreatus* (Jacq.) P. Kumm.] (He, 1991; Xia, 2002)			
				Mushroom [*Schizophyllum commune* Fr.] (Hu et al., 2023)			
				Mushroom [*Auricularia cornea* Ehrenb.] (He, 1991)			
				Mushroom (*Auricularia* sp.) (Li and Li, 1992; Lin, 1993)			
				Mushroom [*Hymenopellis radicata* (Relhan) R.H. Petersen] (Huang, 2019)			
Total 4		17			5	3	2

Classification used for crop plants was derived from the website https://news.cnhnb.com/zywd/detail/441541/.

Mushrooms are listed under cash crops.

Lister (Lister, 1888) reported that the plasmodium of *Badhamia utricularis* could feed upon a number of basidiomycetes and would consume a fruiting body of *Stereum hirsutum* within a few hours. Later, Madelin (Madelin et al., 1975) demonstrated that the plasmodium of *B. utricularis* showed a positive chemotaxis towards a substance produced by *S. hirsutum*. In mushroom cultivation sites, artificial manipulation of the environment provides excessive moisture and large amounts of dead plant material, which favors the growth of myxomycetes. Chung (Chung et al., 1998) summarized the myxomycetes (29 species and 4 varieties) that had been recorded from various edible mushroom cultivation sites in Taiwan, China. The production of edible mushrooms has developed rapidly in China (Fang et al., 2014), and a myxomycete colonization that is not non-pathogenic has affected sustainable and development of a number of different types of mushrooms, including *Lentinula edodes* (Berk.) Pegler (Li et al., 1992), *Pleurotus ostreatus* (Jacq.) P. Kumm (He, 1991), *Auricularia heimuer* F. Wu, B.K. Cui & Y.C. Dai (Li et al., 1992), *Hericium erinaceus* (Bull.) Pers (Lin, 1993), and various other species. This fact implies that slime molds might be a serious problem in some situations. In addition, reports on mushroom diseases caused by slime molds may be overlooked because of a failure in identification. Some mushroom farmers have misunderstand myxomycete colonization and misuse pesticides for prevention and control, which delays the application of truly effective prevention methods and causes unnecessary economic losses (Bao, 2011).

While previous literature on what we refer herein as myxomycete “disease” has predominantly focused on reporting species and cultural or chemical control measures targeting specific species. While these reviews have made significant contributions to the field, they still have limitations in a number of different aspects. First, what is the total number of species of myxomycetes that cause a “disease”, and what is their worldwide distribution? Second, what are the symptoms exhibited by these myxomycete on different crop plants? Third, what measures have been taken to address these “disease” in crop plants? Therefore, the main contribution of this review lies in systematically summarizing myxomycete colonization and the cultural and chemical strategies for the prevention and control of this phenomenon, derived from the published reports that have appeared thus far, which are expected to provide new guidance for research and practice in this field.

## 2 Detection and diagnosis of myxomycetes

Detecting and diagnosing myxomycetes is crucial for understanding their ecology, distribution, and impact. The initial step in detection involves regular field surveys to identify potential habitats for myxomycetes. Habitats such as decaying logs, soil, leaf litter, and mossy areas should be carefully examined for the presence of their fruiting bodies, slime trails, or other characteristic signs of myxomycete activity. The use of a hand lens or magnifying glass can aid in the detection of small or inconspicuous fruiting bodies. Once potential myxomycete habitats have been identified, targeted sampling techniques should be employed to collect specimens for further analysis (Tu et al., 2016). This can involve the use of sterile tools to collect fruiting bodies, slime trails, or soil samples containing plasmodia. The samples should be collected in sterile containers and labeled with relevant information such as location, date, and habitat type.

The initial step in diagnosis involves morphological analysis of the collected samples. This includes examining the shape, color, and texture of fruiting bodies, as well as the structure and behavior of myxamoebae and plasmodia under a microscope (Zhao et al., 2010; Song and Chen, 2024). The use of light microscopy and staining techniques can aid in the determination of characteristic features. For more accurate identification, molecular analysis techniques can be employed. This involves extracting DNA from the samples and amplifying specific genetic markers (Fiore-Donno et al., 2008; Prikhodko et al., 2023) using PCR (polymerase chain reaction). The amplified DNA fragments can then be sequenced and compared to reference databases (https://www.ncbi.nlm.nih.gov/) to identify the species. Based on the results of morphological and molecular analyses, the particular species of myxomycetes can be identified.

## 3 Symptoms of myxomycete colonization

The symptoms of the myxomycete colonization on crops are presented in **Table 1**. We found that myxomycete colonization is primarily manifested on the stems, leaves, and leaf margins of crop plants in the form of fruiting bodies or less commonly plasmodia (Kim et al., 2007, Kim et al, 2009). In some cases, the “disease” expands to cover much of the entire plant. This could potentially interfere with plant photosynthesis, transpiration and respiration by blocking out light and covering stomata (Wang and Pan, 1985; Couch, 1995; Tu et al., 2016; Chen et al., 2021; Shen et al., 2021), causing the loss of plant biomass and thus cultivation value, which will seriously lead to the drying of leaves, the death of the whole plant, and serious shortage of seedlings. The color of the “disease” affecting different crops varies according to the species of myxomycete involved, which can range from white to yellow or orange to black. These could alter the appearance of the crop and make it less aesthetically pleasing.

Most myxomycetes most commonly occur under moist conditions and when wet virtually all types of organic matter provide suitable habitats for these organisms. Myxomycete colonization is sometimes present on a healthy plant. This was the case in one instance in which the myxomycete *Comatricha pulchella* (C. Bab.) Rostaf. were present on the plant, after rinsing or removing the sub-entity with water, the plant tissue at the site where the sub-entity was produced remained healthy (Wang et al., 2014; Tu et al., 2016). Therefore, strictly speaking, because it does not meet the Koch postulate (Ross and Woodward, 2016), a myxomycete colonization cannot be considered as a pathogen. One reason why myxomycete colonization occurs on crop plants in the field may be a result of the spread of humus to leaves as the humus is applied to supplement the soil. Myxomycetes would be expected to be abundant (as amoeboflagellates) in the humus. However, since the spores of myxomycetes are wind-dispersed, they have the potential to land anywhere, including plant surfaces and the soil out of which the plant is growing. As already noted, if the amoeboflagellates ultimately give rise first to plasmodia and then to fruiting bodies, the potential exists for this causing the leaves of crop leaves to have reduced photosynthesis and respiration, thus decreasing their cultivation value (Chen et al., 2021).

## 4 Characteristics of myxomycete colonization

### 4.1 Causes of the “disease”

Myxomycetes are a group of polyphagous eukaryotes characterized by a distinctive life cycle, including a plasmodial stage and fruiting body stage (Hatano and Tazawa, 1968). Plasmodia feed on bacteria, organic substances, fragments of mushroom mycelia, and spores. However, bacteria represent the primary sources of their nutrition (Li et al., 2022). If any kind of organic material is infected with bacteria, or the environment is humid, this provides suitable conditions for myxomycetes. For example, the implementation of no tillage measures after wheat harvest provides a very suitable environment for the growth of myxomycetes. As such, large amount of undeveloped wheat stubble is likely to be affected by these organisms (Filipowicz, 1979; Chen et al., 2021; Dai et al., 2023).

### 4.2 Occurrence regularity

Some myxomycetes can be found, sometimes abundantly, in high-temperature (20–30°C) and high-humidity (about 90%) environments on moist dead grass, in crevices in dead logs, under bark, and on dead leaves and in fertile soils (Everhart et al., 2008; Trevino-Zevallos and Lado, 2020). They are well suited for occurring in places rich in organic matter. Therefore, since the period of July to August each year in much of Asia is a period of high temperature and humidity, this creates conditions for the frequent occurrence of the myxomycete colonization. This “disease” often occurs during hot and rainy seasons other places in the world (Kong et al., 2014). For example, in the process of mushroom cultivation during the growth of the mycelium, when the humidity of the air is too high and not breathable and the temperature also is too high, this makes it easy for a myxomycete colonization to invade the cultivation bag (Bao, 2011). When entering the stage of mushroom emergence, the growth of fruiting bodies requires weak light, high humidity, and a medium temperature environment. In addition, when substances such as bran are added, some bran is not completely decomposed by the tea tree mushroom hyphae. After being dripped with water, water accumulates in the bag, which can easily cause fermentation and acidification of the bran. This environment is suitable for the growth of myxomycetes, so they begin to invade from the bag mouth and gradually affect the mushroom fruiting bodies, causing these to wilt and rot (He, 1991; Li et al., 1992; Lin, 1993).

For this paper, we reviewed the published literature on myxomycetes reported to occur on crop plants reported since 1970, based on the latest 18S rDNA phylogeny (Leontyev et al., 2019) classification of the entire group and http://www.indexfungorum.org/names/Names.asp for current names. In this classification, myxomycetes mentioned in these reports are placed into four orders, five families, and thirteen genera, and twenty-nine species.

### 4.3 Transmission route

The occurrence of myxomycete colonization is closely related to a specific environment. The myxomycetes that occur on plants are probably brought in on soil and culture materials, and their occurrence does not affect the normal growth of crop plants if removed in time. Myxomycete spores are likely transmitted to affected crop plants via wind (Kamono et al., 2009), water (Lindley et al., 2007), insects (Nunes Lemos et al., 2010; Kataoka and Nakamori, 2020), other animals (Trimble, 2021), and human activities. In addition, the spores of myxomycetes can survive inhospitable environments. Wind dispersal of spores is considered to be most important way for myxomycetes to colonize new areas and/or substrates.

## 5 Prevention and control methods of myxomycete colonization

Across the globe, the problem of crop plant security has become more and more prominent. It is known to be significant impact ensuring economic growth, adjusting agricultural structure, impacting the livelihood of farmers, and affecting income (Kang et al., 2017a; Yu et al., 2022). Any increase in a myxomycete colonization can causes problems by reducing crop yield and thus losses in agricultural cultivation. The current research has considered only cultural and chemical control, so this manuscript provides a review of prevention and control strategies for myxomycetes, mainly from two aspects—cultural control and chemical control.

### 5.1 Cultural control

We have summarized cultural control for 11 types of myxomycete colonization for a total of 17 affected crop plants (**Table 3**). Among these, the myxomycete colonization of edible mushrooms accounts for more than 50% of the control method reported. This reflects the prevalence of myxomycete colonization and their potential threat to agricultural production. Due to the dominance of the control method of myxomycete colonization of edible mushrooms, it can be inferred that edible mushrooms may have high economic value in the agriculture or food industry for many human conditions and activities (Ghorai et al., 2009; Shen et al., 2022).

**Table 3:** Cultural control of crops affected by myxomycete colonization.

Affected crops	Disease-causing myxomycetes	Cultural control	References
Mushroom [*Agrocybe chaxingu* N.L. Huang]	*Stemonitis herbatica*	High-quality strains; Sterilization treatment of fruiting bodies; Ventilation and breathability of the mushroom house; Mobile clean water to spray mushroom buds; Mycelium mass should be collected to remove residues	(Liu and Lin, 2003)
Mushroom (*Dictyophora* sp.)	*Stemonitis fusca*	Site selection; Processing of fruiting bodies; Selection of strains; Strengthen bed management	(Bao, 2011)
Mushroom [*Auricularia heimuer* F. Wu, B.K. Cui & Y.C. Dai]	*Physarum pezizoideum* *Physarum utriculare*	Appropriate moisture content when processing fruiting bodies; Use of fresh fruiting bodies, appropriately reduce the proportion of starch-rich exipients such as wheat bran and rice bran; Medium sterilized thoroughly; Strengthen ventilation and reduce air humidity	(Li et al., 1992)
Mushroom [*Lentinula edodes* (Berk.) Pegler]	——	Remove waste and ground soil layers; Reduce the temperature and humidity of the mushroom house and maintain ventilation; Nutrien and mushroom sheds exposed to the sun	(Kong et al., 2014)
Dendrobe [*Dendrobium officinale* Kimura et Migo]	*Fuligo septica* *Comatricha pulchella*	Control the moisture of the matrix	(Tu et al., 2016)
Sweet potato [*Ipomoea batatas* (L.) Lam.]	*Fuligo gyrosa*	Ventilation and light transmission; Reduce temperature and humidity	(Su, 1987)
Mushroom *Auricularia* sp. [*Lentinula edodes* (Berk.) Pegler] [*Hericium erinaceus* (Bull.) Pers.]	*Physarum* sp. *Tubifera* sp. *Stemonitis splendens*	Shovel away the diseased strains and substrate; Increase light	(Lin, 1993)
Peanut [*Arachis hypogaea* Linn.]	*Fuligo gyrosa*	Extinguish the stubble in the whole land; Apply fully rotted organic fertilizer; Drainage in time after rain; Reduce field humidity	(Liu et al., 2007; Zhao et al., 2010; Bao, 2011; Wang et al., 2014, Wang et al, 2016)
Wheat [*Triticum aestivum* L.]	*Physarum cinereum*	Extinguish the stubble in the whole land; Apply fully rotted organic fertilizer; Drainage in time after rain; Reduce field humidity	(Liu et al., 2007; Zhao et al., 2010; Bao, 2011; Wang et al., 2014)
Foxtail millet [*Setaria italica* (L.) P. Beauv.]	*Fuligo gyrosa*	Deeply turn the stubble and remove weeds in the field; Apply rotten organic fertilizer; Excavate a drainage ditch	(Chen et al., 2021)
Mushroom (*Auricularia* sp.)	*Physarum pezizoideum*	Improve the environmental conditions of field; Reduce air humidity; Extreme pH and extreme osmotic pressure	(Li and Li, 1992)
Ginseng [*Panax ginseng* C. A. Meyer]	*Stemonitis herbatica*	Field inspection; Remove diseased fruiting bodies	(Bai and Jiang, 1988)
Mushroom [*Hymenopellis radicata* (Relhan) R.H. Petersen]	——	High-quality fruiting body; Mycelium mass is fully cooked; Appropriate water content of the soil-covered nutrient; Appropriate temperature, humidity and CO_2_ concentration; Use clean water sources; Keep the mushroom house clean	(Huang, 2019)
Dendrobe [*Dendrobium officinale* Kimura et Migo]	*Fuligo septica* *Comatricha pulchella*	Pruning in batches; Strengthen fertilizer and water management; Suitable shed room Ventilation; Controlled watering, humidity	(Xie et al., 2017; Shen et al., 2021)
Chilli [*Capsicum annuum* Linn.]	——	Farmer’s fertilizer is fully decomposed; Clear the groove and drain the stains; Reduce field humidity	(Wang and Pan, 1985)

From the perspective of the statistical measures given in the table as a whole, for edible fungi one should adhere to the principle of “prevention first, comprehensive prevention and control”. The key to preventing and controlling myxomycete colonization is to improve the cultivation environment, control the moisture content of culture materials and deal with the incidence area in a timely manner (He, 1991; Lin, 1993; Kong et al., 2014; Li et al., 2017).

Cultural practices could include intercropping, crop rotation, and balanced doses of fertilizer, the specific recommendations often vary depending on the agro-ecological conditions, soil type, climate, and pest and disease pressure in a particular region. In general, selecting crops with complementary nutrient requirements and growth habits can enhance biodiversity and soil fertility, which may indirectly affect myxomycete populations (Liu et al., 2007; Zhao et al., 2010; Bao, 2011; Wang et al., 2014, Wang et al, 2016). For instance, legumes (such as beans and peas) can be intercropped with cereals (like wheat and maize) as they fix nitrogen from the air, which benefits the cereals (Wahbi et al., 2016). In crop rotation, rotating grains with legumes or vegetables can break disease cycles and improve soil structure (Ball et al., 2005; Ghosh et al., 2020). As for the role of balanced fertilizer doses in disease reduction, the key lies in maintaining optimal nutrient availability for plant growth while avoiding nutrient imbalances that can stress plants and make them more susceptible to diseases (Marouelli et al., 2015). Excessive nitrogen, for example, can promote vegetative growth but also make plants more vulnerable to fungal diseases (Jeon, 2019). By precisely applying the right mix of nutrients, including macronutrients (nitrogen, phosphorus, potassium) and micronutrients (like zinc and iron), we can promote healthy plant growth, strengthen the plant’s immune system, and thereby reduce disease pressure.

For cultivated crops such as peanuts, wheat, ginseng, chili peppers, and *Dendrobium officinale*, farmers should conduct regular field inspections. The fields cultivated should be incorporated into the local farming system by completing the stubble soon after harvesting, applying fully decomposed organic fertilizer, and adopting formula fertilization technology. Also, excess water should be drained soon after the rain to prevent the field from being too wet (Wang and Pan, 1985; Bai and Jiang, 1988; Wang et al., 2014; Shen et al., 2021).

### 5.2 Chemical control

Chemical control is known to result in environmental contamination of the biosphere and has become a debatable concern globally. Moreover, most methods are expensive and environmentally unfriendly (Naidu et al., 2021; Tudi et al., 2021). However, current chemical control measures that involve the spraying of traditional chemicals or antibiotics can have an effect on myxomycete colonization. Xing (Xing et al., 2011) selected low toxic and pollution free chemical reagents to conduct indoor toxicity tests to determine inhibition to myxomycetes; Then through indoor bioassay results to select the reagents with small effect on the growth of *Pleurotus ostreatus*. Chemical control that have been used for nine types of myxomycete “disease” in a total of 16 affected crop plants is summarized in **Table 4**.

**Table 4:** Control of crops affected by myxomycete colonization using synthetic chemicals.

Affected crops	Disease-causing myxomycetes	Chemical control		References
		Reagent	Dilution times	
Mushroom [*Pleurotus ostreatus* (Jacq.) P. Kumm.]	——	Bouilliebordelaise		(Xia, 2002)
Edible fungi	——	Ludanlan sophora flavescens + Salicylic acid	1000 + 300	(Xing et al., 2011)
		Clolrimazole suppositories	1000	
		Ludanlan sophora flavescens	1000	
		Salicylic acid	300	
Mushroom [*Cyclocybe aegerita* (V. Brig.) Vizzini]	——	Penicillin + 0.15% Chloramphenicol	200	(Guan, 2004)
Mushroom [*Agrocybe chaxingu* N.L. Huang]	*Stemonitis herbatica*	50% Carbendazim		(Liu and Lin, 2003)
		70% Thiophanate-methyl +Streptomycin	100~200	
Mushroom [*Agrocybe chaxingu* N.L. Huang]	*Stemonitis pallida*	Bouilliebordelaise Bouilliebordelaise+ Streptomycin		(Liu, 2007)
Mushroom (*Dictyophora* sp.)	*Stemonitis fusca*			
Mushroom (*Dictyophora* sp.)	*Stemonitis fusca*	Bouilliebordelaise Bouilliebordelaise+ streptomycin	300、500 300、500	(Liu et al., 2007)
Peanut [*Arachis hypogaea* Linn.] Wheat [*Triticum aestivum* L.]	*Fuligo gyrosa* *Physarum cinereum*	Bouilliebordelaise Streptomycin 36% Thiophanate-Methyl 50% Benomyl 80% Mancozeb 30% Hymexazol	200 100~200 500 1000 600~1000 1000	(Lu, 2001; Liu et al., 2007; Bao, 2011; Wang et al., 2014)
Peanut [*Arachis hypogaea* Linn.] Foxtail millet [*Setaria italica* (L.) P. Beauv.] Sesame [*Sesamum indicum* Linn.] Corn [*Zea mays* L.]	*Fuligo gyrosa*	50% Carbendazim FengQia^®^GenBao 43% Tebuconazole Bouilliebordelaise 50% Benomyl 80% Mancozeb 36% Thiophanate-Methyl 30% Hymexazol	800 600~800 5000~7000 200 1000 600~1000 500 1000	(Li, 2012; Wang et al., 2016; Chen et al., 2021)
Dendrobe [*Dendrobium officinale* Kimura et Migo]	*Comatricha pulchella*	lime		(Shen et al., 2021)
Chilli [*Capsicum annuum* Linn.]	——	0.25–0.3% Bouilliebordelaise Carbendazim Thiophanate-methyl	800 1000	(Wang and Pan, 1985)
Mushroom *Auricularia* sp. [*Lentinula edodes* (Berk.) Pegler] [*Hericium erinaceus* (Bull.) Pers.]	*Physarum* sp. *Tubifera* sp. *Stemonitis splendens*	limewater		(Lin, 1993)

Chemicals were applied by spraying. “%” refers to the mass percentage; “+” refers to a combination.

The corresponding prevention and control strategies of the effective reagents reported thus far are outlined in **Table 4**. These data indicate that the different combinations of reagents and dilution ratios reflect the precise management tailored for different types of crops and edible mushrooms. The reason for this may be that regional differences have caused myxomycete colonization to display different patterns of occurrence on crops. Judging from the overall situation, the three most frequently used reagents are Bouilliebordelaise, Thiophanate-methyl, and Streptomycin, indicating that they show a certain degree of universality and effectiveness in controlling the “disease” on crops and edible mushrooms. However, in the future, chemical control methods should not be limited to specific types of reagents but also should include broader chemical control methods, especially those widely used synthetic fungicides.

## 6 Conclusions and future perspectives

Myxomycetes prefer humid conditions and are commonly occur in association with all kinds of organic material, including living plants. When myxomycete fruiting bodies are found on a living plant, the initial impression is that they are pathogenic. However, this is not the case. Nevertheless, myxomycetes can indeed affect the normal growth of plants by reducing photosynthesis and respiration. Most of the time it is inconsequential, but in some instances it is not. In time the spores produced by the fruiting bodies can adhere to other healthy plants as a result of being spread by wind and rain. Occurrence of myxomycete colonization is closely related to specific set of environmental conditions that include large amounts of decaying organic matter and the presence of a plentiful supply microorganisms, especially bacteria). Because of this, the “disease” has an obvious regional occurrence.

This review summarizes the current examples of myxomycetes affecting plant (including mushroom) growth and effective prevention and control measures, including cultural control and chemical control. Myxomycete colonization has caused serious economic losses to China’s local edible fungus industry. Therefore, we sincerely hope that more scholars will pay attention to myxomycete colonization in the future. For example, in production the relative air humidity in the growing shed can be controlled by constructing a suitable shed, maintaining proper ventilation in the shed, controlling watering, and sprinkling a reagent around the seedbed, thus controlling the occurrence of myxomycete colonization. However, we still face many challenges in the mechanism of myxomycete colonization and their understanding of the environment and ecosystem. Scientific questions that need to be answered in the future include: (1) Is there genetic exchange between the cultivation matrix of myxomycetes and mushrooms? What is the specific mechanism? (2) Will the bacteria carried by myxomycete cause plant diseases?

Future research on myxomycete “diseases” should focus on elucidating the genetic and molecular mechanisms underlying the “pathogenicity” of these organisms. Genomics and proteomics studies can provide insights into their virulence factors and how they interact with host crop plants. In addition, ecological studies examining the role of myxomycetes in ecosystems and their interactions with other organisms are needed to develop more targeted and sustainable management strategies. Current management strategies for myxomycete “diseases” face several challenges. One major concern is the development of resistance to chemical reagents, which threatens the effectiveness of traditional chemical control methods. Furthermore, the use of chemical reagents can have negative environmental impacts, including water pollution and disruption of ecological balance. Cost-effective and environmentally friendly alternative control methods need to be explored. Additionally, the variability in “disease” incidence and severity across different regions and conditions poses a challenge in developing universally applicable management strategies.

In summary, future research should have the objective of providing a deeper understanding of the biology and ecology of myxomycetes, while management strategies should focus on developing sustainable and cost-effective alternatives to chemical reagents.

## Author contributions

ZZ: Conceptualization, Data curation, Software, Writing – original draft. CZ: Software, Writing – review & editing. YL: Project administration, Supervision, Writing – review & editing. SS: Conceptualization, Formal analysis, Writing – review & editing. PL: Funding acquisition, Project administration, Resources, Writing – review & editing.
